# Distinctive waves of innate immune response in the retina in experimental autoimmune encephalomyelitis

**DOI:** 10.1172/jci.insight.149228

**Published:** 2021-06-08

**Authors:** Andrés Cruz-Herranz, Frederike C. Oertel, Kicheol Kim, Ester Cantó, Garrett Timmons, Jung H. Sin, Michael Devereux, Nicholas Baker, Brady Michel, Ryan D. Schubert, Lakshmisahithi Rani, Christian Cordano, Sergio E. Baranzini, Ari J. Green

**Affiliations:** 1 Weill Institute for Neurosciences, Department of Neurology, University of California San Francisco, San Francisco, California, USA.; 2 Experimental and Clinical Research Center (ECRC), Max-Delbrück-Centrum for Molecular Medicine, and; 3 NeuroCure Clinical Research Center (NCRC), Charité – Universitätsmedizin Berlin, corporate member of Freie Universität Berlin, Humboldt-Universität zu Berlin, and Berlin Institute of Health, Berlin, Germany.; 4 Department of Ophthalmology, University of California San Francisco, San Francisco, California, USA.

**Keywords:** Neuroscience, Innate immunity, Multiple sclerosis, Neurodegeneration

## Abstract

Neurodegeneration mediates neurological disability in inflammatory demyelinating diseases of the CNS. The role of innate immune cells in mediating this damage has remained controversial with evidence for destructive and protective effects. This has complicated efforts to develop treatment. The time sequence and dynamic evolution of the opposing functions are especially unclear. Given limits of in vivo monitoring in human diseases such as multiple sclerosis (MS), animal models are warranted to investigate the association and timing of innate immune activation with neurodegeneration. Using noninvasive in vivo retinal imaging of experimental autoimmune encephalitis (EAE) in CX3CR1^GFP/+^–knock-in mice followed by transcriptional profiling, we are able to show 2 distinct waves separated by a marked reduction in the number of innate immune cells and change in cell morphology. The first wave is characterized by an inflammatory phagocytic phenotype preceding the onset of EAE, whereas the second wave is characterized by a regulatory, antiinflammatory phenotype during the chronic stage. Additionally, the magnitude of the first wave is associated with neuronal loss. Two transcripts identified — growth arrest–specific protein 6 (*GAS6*) and suppressor of cytokine signaling 3 (*SOCS3*) — might be promising targets for enhancing protective effects of microglia in the chronic phase after initial injury.

## Introduction

Microglia are the resident immune cells of the CNS and are part of the innate immune system (1). During inflammation, microglia recruit lymphocytes and peripheral monocytes. These cells also produce inflammatory cytokines, proteases, and free radicals, which all contribute to CNS damage. (1) Conversely, microglia also maintain CNS integrity by removing apoptotic cells, providing trophic support, and potentially enhancing remyelination (2). In patients with multiple sclerosis (MS), distinct subsets of microglia have been suggested to play both beneficial and destructive roles (2–4). The heterogeneity of microglial phenotypes at different time points and in different CNS regions further diversify their roles (5, 6). Thus, animal studies that model human MS pathology are warranted to investigate the association of microglial activation with damage and to explore microglial phenotypes in distinct disease stages.

Myelin oligodendrocyte glycoprotein–induced (MOG_35–55_–induced) experimental autoimmune encephalomyelitis (EAE) is a widely studied animal model of MS (7). In mice, MOG_35–55_ EAE induces a myelin targeted adaptive immune-led response with a chronic, nonremitting phenotype characterized by an ascending myelitis and optic neuritis. EAE lesions demonstrate perivenous inflammatory mononuclear infiltrate with gliosis, confluent plaques of demyelination, and relative axonal sparing (8). Up to 90% of EAE mice develop retinal and optic nerve lesions, making the afferent visual system an important location in which to study microglial changes in neuroinflammation (3, 8, 9).

It has been suggested that microglial activation fuels inflammation and recruits additional immune cells from the periphery (3). However, whether microglial activation also plays a direct role in retinal neurodegeneration or neuronal repair has remained unclear (8). For CNS white matter, it was recently suggested that a switch from a proinflammatory to a proregenerative microglial phenotype coincides with the initiation of remyelination and repair (10, 11); however, their role in unmyelinated retina and gray matter is still undetermined (2, 8). These reports of dynamic microglial changes have further been challenged by data suggesting (a) a stable number of retinal microglia over the course of EAE or (b) no effect of microglial depletion on the severity of EAE (12, 13). As a consequence, the true dynamics of microglia remain uncertain.

Prior animal studies performing cross-sectional histopathological analyses at specific time points are united by the limited access to relevant tissue, leading to conflicting results due to different time points and pseudolongitudinal analyses. Therefore, noninvasive techniques, which can longitudinally monitor neuroinflammation with close-to-cellular resolution, can add to our understanding. The retina is an accessible part of the CNS for in vivo imaging and enables the noninvasive investigation of dynamic processes with high-resolution, nontoxic, light-based methods such as optical coherence tomography (OCT) and confocal scanning laser ophthalmoscopy (CSLO). In CX3CR1^GFP/+^–knock-in mice, microglia, macrophages, monocytes, DCs, and NK cells express green fluorescent protein (GFP), which can be imaged through CSLO (14), without immunologic alterations (15). The combined use of longitudinal retinal OCT and CSLO in EAE of CX3CR1^GFP/+^–knock-in mice allows for the dynamic study of innate immune activation in the retina and, thereby, allows for more direct and longitudinal assessment of innate immune cell response compared with traditional histopathology.

We aimed to longitudinally characterize the association of microglial activation with disease severity and neuroaxonal damage, as well as to identify the transcriptomic signature of retinal innate immune cells at distinct time points in EAE.

## Results

Despite microglia being the most motile retinal innate immune cells, their activation in the retina has been only partially described in EAE; the number of resident microglia increases early (around 7 days postinfection [dpi]) (8). Microglia then spread out, become activated, and proliferate quickly, reaching their peak count around 11–15 dpi. Their number remains elevated until 60 dpi (8, 9, 13, 16). Whether microglial activation plays a direct injurious or protective role in retinal neuroinflammation and how these processes are related in time has remained unclear (8). As a consequence, we first investigated the true dynamics of retinal innate immune cells in EAE.

### Two distinct waves of retinal innate immune cell activation in EAE.

After immunization with MOG_35–55_ peptide in complete Freund’s adjuvant containing mycobacterium tuberculosis and with 200 ng pertussis toxin, 7 EAE mice developed neurological symptoms (onset: [median (min–max)]: 11 dpi [8–15 dpi]) and were followed for 56 days after induction (Supplemental Figure 1A; supplemental material available online with this article; https://doi.org/10.1172/jci.insight.149228DS1).

**Figure 1:**
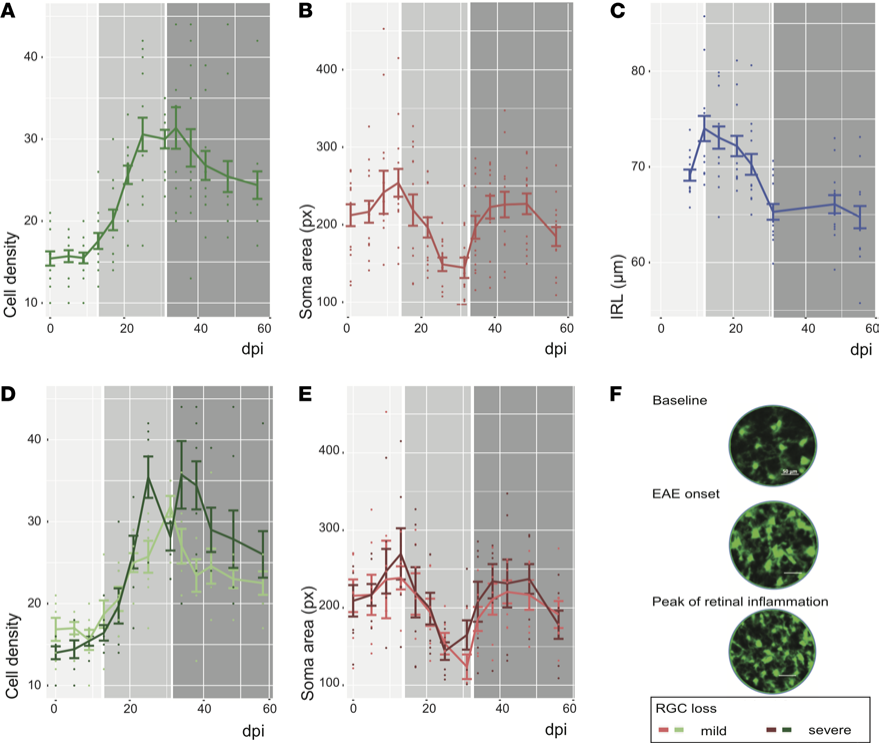
Longitudinal in vivo imaging in EAE. (**A** and **B**) Preonset (light gray 0–13 dpi), the cell density (**A**) and soma area (**B**) of CX3CR1 GFP^+^ cells increase. Whereas the cell density of CX3CR1 GFP^+^ cells further increases during the acute phase (13–30 dpi; medium gray) and decreases in the chronic phase (31–56 dpi; dark gray), the soma area of CX3CR1 GFP^+^ cells decreases in the acute phase and then peaks again the chronic phase. (**C**) The IRL thickness increases before onset, drops dramatically during the acute phase, and stabilizes in the chronic phase of EAE. (**D** and **E**) Eyes with severe RGC loss compared with eyes with mild RGC loss at 56 dpi showed a higher cell density of CX3CR1 GFP^+^ cells 25 dpi and 38 dpi (**D**) and a less relevant drop of CX3CR1 GFP^+^ cell soma area (**E**). (**F**) Cell density and soma area changes of CX3CR1 GFP^+^ cells are qualitatively visible by CSLO (please note: images falsely colorized). For **A**–**E**, data are shown as mean ± SEM; experiment included eyes of 7 mice.

During the inner retinal layer (IRL) swelling preonset (Figure 1C), the CX3CR1 GFP^+^ cell density (0 dpi: 15.4 ± 3.3 cells per selected area; 13 dpi: 17.6 ± 3.7 cells) and soma area (0 dpi: 212.2 ± 52.7 pixel [px]; 13 dpi: 254.0 ± 67.1 px) measured by CSLO increased only slightly, without reaching significance. However, cell density increased (31 dpi: 30.0 ± 4.2, *P <* 0.001) and soma area decreased (31 dpi: 144.4 ± 49.2 *P <* 0.001) significantly in the acute inflammatory and neurodegenerative phase thereafter (13–31 dpi, Figure 1, A–C). Conversely, the density of CX3CR1 GFP^+^ cells decreased (56 dpi: 24.4 ± 6.0, *P =* 0.003) and the soma area increased (56 dpi: 184.7 ± 45.8, *P <* 0.001) again in the chronic phase (31–56 dpi) with stabilized neuronal content. Next, we investigated if neuroaxonal loss was associated with early innate immune activation.

### Early innate immune activation predicts ultimate neuronal cell loss.

At 56 dpi, the retinal ganglion cell (RGC) count measured by the average Brn3a^+^ cell count was 434.5 ± 117.8 per retinal field. Eyes with severe RGC loss (RGC count/field ≤ median of 460.2) showed a higher peak of CX3CR1 GFP+ cell density 25 dpi (β = 10.0, standard error [SE] = 3.5, *P =* 0.020) and 38 dpi (β = 10.6, SE = 3.8, *P =* 0.016) compared with eyes with mild RGC loss (RGC count > median; Figure 1, D–F). Eyes with mild RGC loss had significantly smaller CX3CR1 GFP^+^ cells around the peak of retinal inflammation (38 dpi) compared with eyes with severe RGC loss (β = –123.0, SE = 20.0, *P <* 0.001). This suggests that the magnitude and cellular composition of the inflammatory response impacts neuronal survival but is not sufficient to explain the pronounced differences observed. The observation of 2 distinct peaks separated by a trough identified for us 2 separate waves *(*~15 dpi and ~40 dpi*)* of innate immune cell activation. We therefore hypothesized that different innate immune cell populations with distinct expression profiles contribute to the so far unexplained observed differences, and we embarked to characterize the transcriptional profile of innate immune cells during the course of EAE.

### Transcriptional profiling during EAE is most variable in microglia.

To characterize retinal innate immune cells, we implemented a second EAE experiment. We performed single-cell RNA sequencing (scRNA-seq) of retinal CD11b^+^ cells obtained at 0 (not immunized), 6 (preonset), and 25 dpi (peak of retinal inflammation). Mice at 0 and 6 dpi were free of clinical signs. For 25 dpi, 13 of 20 EAE mice developed neurological signs (Supplemental Figure 1B).

**Figure 2:**
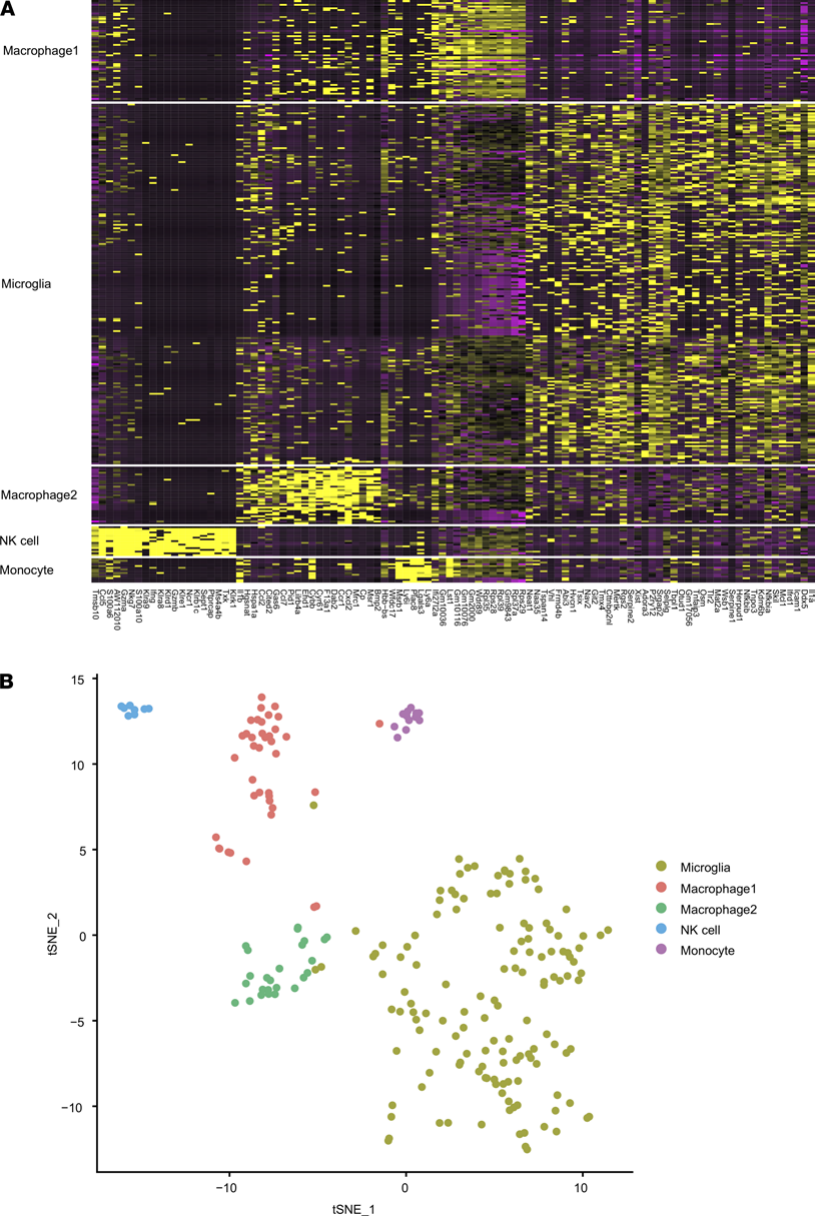
Transcriptomic profile of retinal CD11b^+^ cells during the course of EAE. (**A**) Heatmap of transcriptomic profile in different CD11b^+^ cell types. (**B**) t-SNE plot of CD11b^+^ immune cell clusters based on genetic markers from **A** (pooled from 0, 6, and 25 dpi).

A total of 280 CD11b^+^ retinal cells were sorted: at day 0 (i.e., nonimmunized controls, *n =* 121 cells), at day 6 (*n =* 118 cells), and at day 25 (*n =* 41 cells) after immunization. Sorted cells were then clustered according to their transcriptomic profile, and different cell subsets were identified (Figure 2 and Table 1). The full data set and raw data are accessible on Gene Expression Omnibus (GEO; GSE173378; https://www.ncbi.nlm.nih.gov/geo/query/acc.cgi?acc=GSE173378). We identified microglia, macrophages, NK cells, and activated monocytes by expression of specific molecular markers (Table 1). The different CD11b^+^ cell type fractions changed only marginally over the course of EAE, with a slight, transitory increase of macrophages prior to EAE onset (6 dpi, n.s.). Whereas macrophages, NK cells, and monocytes showed a rather homogenous expression profile, microglial profiles were more heterogenous, especially over to course of EAE — suggesting an innate immune cell shift mainly in the microglial compartment.

### Transcriptional profiling reveals a switch of microglial phenotype from injurious to protective between peak of disease and the later chronic condition.

Prior studies have not clarified the role of early microglia in EAE. One study suggested that depletion of microglia before induction of EAE was associated with worse clinical disease but, paradoxically, no greater injury — while another study suggested a beneficial effect of microglial depletion shortly after EAE induction (12, 17). These seemly conflicting findings necessitate further characterization of innate immune response in the retina with EAE. We theorized that an important way to distinguish the different subpopulations could be an assessment of their transcriptional profiles.

**Figure 3:**
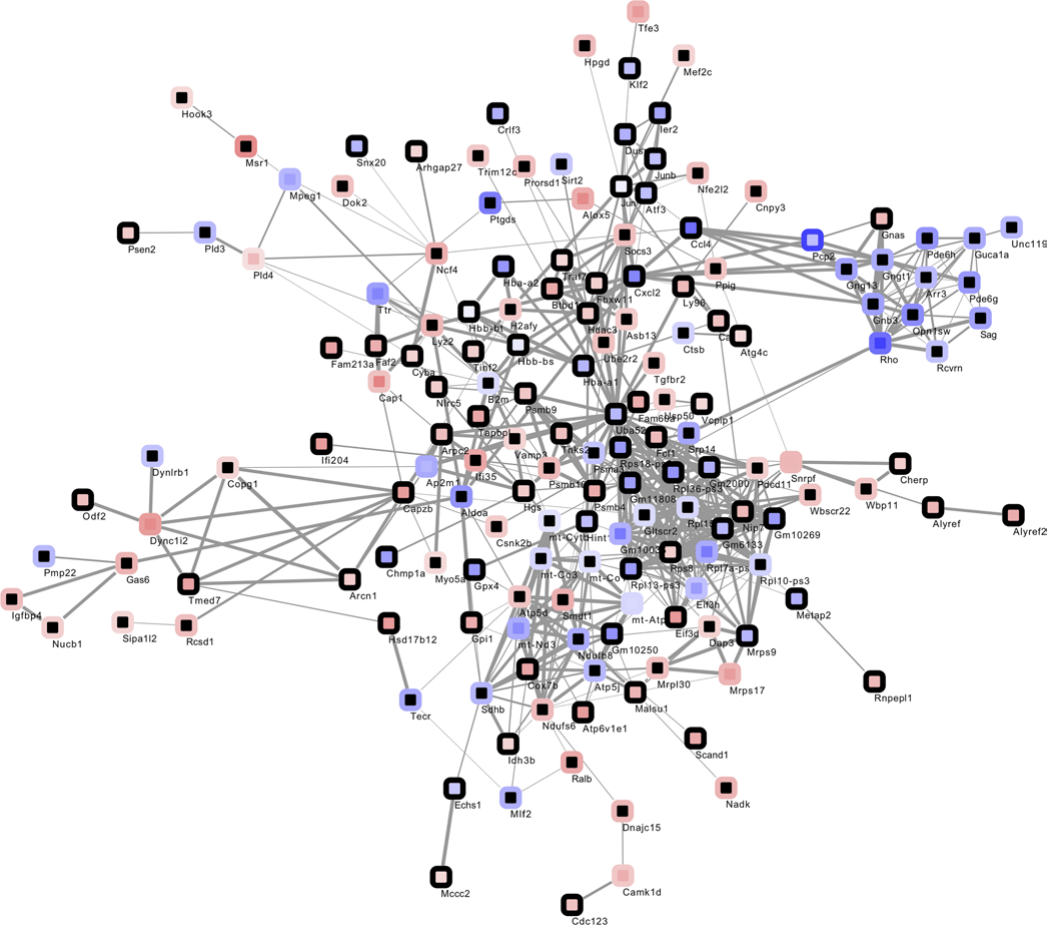
Interaction network of transcripts showing changed expression at 6 dpi (center) or 25 dpi (rim) compared with baseline. Node color mapped continuously dependent on log_FC_ from blue for reduced expression (6 dpi, log_FC_ –1.92; 25 dpi, log_FC_ –2.78) to red for enhanced expression (6 dpi, log_FC_ 1.92; 25 dpi, log_FC_ 2.78); black signifies absence of significant change at respective time point. Edge widths mapped continuously by interaction score from 0.4 (minimal width) to 1 (maximal width).

We therefore characterized the expression profiles of microglia at 6 dpi (before onset; total cell count, *n =* 66) and 25 dpi (peak of retinal inflammation; total cell count, *n =* 28) compared with 0 dpi (total cell count, *n =* 80), and we generated interaction networks and enrichment analyses using STRING and STRING ENRICHMENT by Cytoscape (Figure 3 and refs. 18, 19). When performing pathway enrichment analyses in transcripts, which were at least 2-fold upregulated, our data show an enrichment of inflammatory microglial pathways at 6 dpi, such as neutrophil granulation and reactive oxygen species (ROS) production, as well as CLEC7A (Dectin-1) signaling, a pathway inducing the production of various proinflammatory cytokines and chemokines, including TNF-α and IL-1β, and triggering phagocytosis and further ROS production (Table 2 and refs. 20, 21). At 25 dpi, these pathways were not further enriched; in contrast, we found an enrichment of antiinflammatory pathways such as the biosynthesis of resolvins, which are necessary for the metabolism and degradation of ROS (22). The switch to a noninflammatory, protective role of microglia at 25 dpi was further supported by the downregulation of inflammatory pathways such as antigen-processing cross-presentation and microglial polarization pathways (β-catenin–independent WNT signaling, planar cell polarity/convergent extension [PCP/CE] pathway), which are characteristic for proinflammatory and phagocytic microglia. (23, 24).

It remains under debate whether a downregulation of harmful or an upregulation of protective microglial pathways can, when timed correctly, achieve an attenuation of EAE in mice — or maybe even of human MS as a potential therapeutic target. So far, the most promising target in the literature, which was reported as elevated at 25 dpi in our EAE study, is growth arrest–specific protein 6 (*GAS6*). In microglia, *GAS6* is described to improve the clearance of apoptotic cell bodies and motility (25). However, it is also involved in survival of neuronal and other glial cells, making an off-target cell effect less concerning. In EAE, the administration of *GAS6* leads to reduced demyelination and inflammation, as well as enhanced remyelination (25). A study in cuprizone-induced demyelination, a toxic demyelination model, confirmed the positive effects of *GAS6*, showing a better clearance of debris, more remyelination, and maturation of oligodendrocytes and a higher number of myelinated axons (26, 27). Although the microglial expression of *GAS6* under healthy conditions is lower in humans than in mice, descriptive studies showed that MS patients with a higher *GAS6* concentration in the cerebrospinal fluid (CSF) underwent shorter and less severe MS flares (28). Therefore, the administration or pharmacological enhancement of *GAS6* — or an earlier uptake in expression — might reduce tissue damage and disability accrual also in MS. Another promising target described in the MS literature and enhanced at 25 dpi might be a suppressor of cytokine signaling 3 (*SOCS3*), an inhibitor of JAK/STAT and leptin pathways in microglia, which could reduce the toxic inflammatory response during relapse — probably by regulating IL-6 (29, 30). However, caution is warranted, since *SOCS3* also inhibits the production of leukemia inhibitory factor (LIF), a factor involved in survival and differentiation of oligodendrocytes (31). Equal care should be taken, when considering Arachidonate 5-lipoxygenase (*ALOX5*) as a therapeutic target, which was elevated at both 6 and 25 dpi in our data set. *ALOX5* seems to be involved in both the production of proinflammatory chemotactic factor and antiinflammatory resolvins during different stages of EAE, which explains the inconsistencies of previous studies on *ALOX5*’s effects in EAE and underlines the importance of exactly timed therapies when targeting microglia during the disease course (32–34).

## Discussion

The importance of the innate immune cells in the pathological processes that unfold in response to inflammatory demyelinating injury to the CNS is unambiguous. Both microglia and infiltrating innate immune cells (i.e., monocytes, macrophages, and neurophils) play a role in disease processes, and they likely participate in injury as well as protection (2, 10, 35, 36). The role of microglia and other innate immune cells is better understood in the white matter — the primary site of adaptive immune infiltration and injury. However, damage to the gray matter is critical in mediating permanent irrecoverable injury in MS and EAE (37, 38). Therefore, it is crucial to understand the dynamics of the retinal innate immune response and determining whether innate immune cells play their roles for mediating and protecting against injury simultaneously or at different times — even via separate populations of cells.

In this study, using longitudinal imaging and single-cell transcriptional profiling, we show that the retinal innate immune response in EAE takes place in 2 distinct waves: the early microglial response before onset (6 dpi), which is associated with subsequent neuroaxonal damage and is characterized by a pathogen response profile, and a later response (25 dpi), which is characterized by a more neuroprotective profile. Through longitudinal monitoring of the density of a targeted population of cells, our data suggest that these distinct effects are mediated via different populations. Understanding the dynamics of injurious and protective microglial activation in EAE is a critical step to distinguishing and finally modulating microglial activity (i.e., enforcing protective and inhibiting harmful effects).

Prior important descriptive analysis of retinal histopathology associated with in vivo OCT already inferred a possible direct effect of microglia on neuroaxonal damage. In addition to evaluating other cell types, histopathological evidence showed an early increase of microglia, and in separate animals in vivo, OCT showed later IRL thinning as a metric of neuroaxonal injury (8). By combining concurrent OCT and CLSO, we were able to detail the dynamics of microglial response. We confirmed an early increase in microglial numbers preceding the onset of neuroaxonal damage and established an indirect association between the number of innate immune cells at peak of disease and later RGC loss. Thus, our data confirm that, even in a myelin-devoid area of the CNS like the retina, early immune activation — particularly of innate immune cells — help mediate the inflammatory response and have downstream injurious effects on neurons in EAE.

In the case of white matter, research indicates that the transition from a proinflammatory to a proregenerative microglial population is necessary to initiate remyelination and repair (2, 35, 39). Our CSLO data show a reduced cell density, and soma area of retinal microglia shown around 30 dpi suggests a necroptosis and repopulation of microglia. These results, thereby, suggest that the switch from a proinflammatory to a proregenerative microglial population is not solely based on the interaction of microglia with myelinated axons but that it is also present in unmyelinated areas such as the retina. Therefore, this phenotype switch is not specific to remyelination but likely affords a milieu that is directed generally toward repair.

The transcriptomic profile of microglia prior to onset of EAE (6 dpi) showed the increased expression of various genes involved in immune response underlining the involvement of microglia in the acute inflammatory process. These results are in line with an inflammation-enhancing and phagocytic microglial phenotype preceding disease onset. At the peak of retinal inflammation (25 dpi), the upregulation of these genes was already reversed in parts; multiple genes involved in antiinflammatory response were upregulated and polarization was downregulated, pointing toward a protective microglial function in the later stage of disease (40). Nevertheless, the purpose of a proregenerative microglia in the absence of myelin warrants further investigations.

It should be noted that OCT is limited in rodents to the central retina and lacks cellular resolution of RGCs. Also, although the CSLO and histological measurements matched well, they are still not fully congruent. Finally, our study lacked scRNA-seq analyses (a) at 15 dpi to show microglial changes at the peak of clinical signs, (b) at 50 dpi to show a persistence of the proregenerative microglial subsets, and (c) of optic nerve microglia. Future work could prove direct neurodegenerative effects and regenerative potential of microglia by depleting microglia at different time points in EAE (41).

To conclude, our data suggest that microglia play an important role in both retinal neurodegeneration and regeneration during EAE and that a phenotype switch is associated with the beginning of regeneration and repair. Further studies are necessary to understand the pathophysiology of different microglial functions during the EAE disease course and their potential value as therapeutic targets.

## Methods

### Experimental design

**Figure 4:**
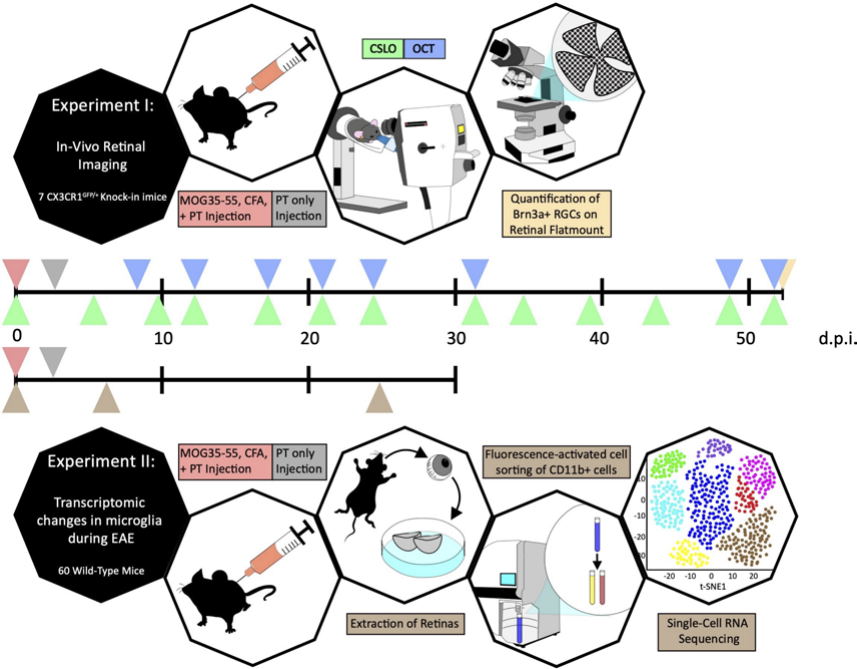
Experimental design. Timelines for experiment I (first row) and experiment II (second row) with color-coded triangles marking time points for MOG_35–55_, CFA, and pertussis toxin (PT) injection (red), PT injection (gray), CSLO imaging (green), OCT imaging (blue), quantification of retinal ganglion cells on flatmounts (yellow), and extraction of retina for FACS and scRNA-seq analyses (brown).

First, we monitored retinal changes during EAE through OCT (retinal thickness) and CSLO (density and morphology of innate immune cells). We then compared the expression profiles of retinal innate immune cells at different time points of EAE through scRNA-seq (Figure 4).

### In vivo retinal monitoring in EAE

#### Mice.

For experiments involving in vivo retinal imaging, CX3CR1^GFP/+^–knock-in mice were fully backcrossed into C57BL/6J. We confirmed the absence of an RD8 mutation (associated with hereditary macular degeneration). In CX3CR1^GFP/+^–knock-in mice, microglia, macrophages, monocytes, and NK cells express GFP, which can be imaged through CSLO (14), without any immunologic alterations (15).

#### Induction and clinical scoring of MOG_35-55_ EAE.

Seven female mice at 10 weeks of age were immunized s.c. with 100 μg of MOG_35–55_ peptide (Genemed Synthesis) in complete Freund’s adjuvant containing 400 μg *Mycobacterium tuberculosis* H37Ra (Difco Laboratories). Mice received 200 ng pertussis toxin (List Biological) by i.p. injection at the time of and after immunization. We monitored clinical scores, as described before (42).

#### Anesthesia.

For retinal imaging, mice were anesthetized by mask inhalation of isoflurane vaporized at concentrations of 1.5% (2 L/min), and their pupils were dilated with 1% tropicamide ophthalmic solution (Akorn).

#### OCT.

On 8, 12, 16, 21, 31, 48, and 55 dpi, after CSLO scanning and without moving the mouse, we performed volume OCT scans of the IRL (combination of the RNFL and the ganglion cell plus the inner plexiform layers) as previously described (42, 43). Scans consisted of 25 B-scans (2-dimensional images) recorded in high-resolution mode and rasterized from 30 averaged A-scans (1-dimensional scans).

#### Reliability of CSLO measurements.

**Figure 5:**
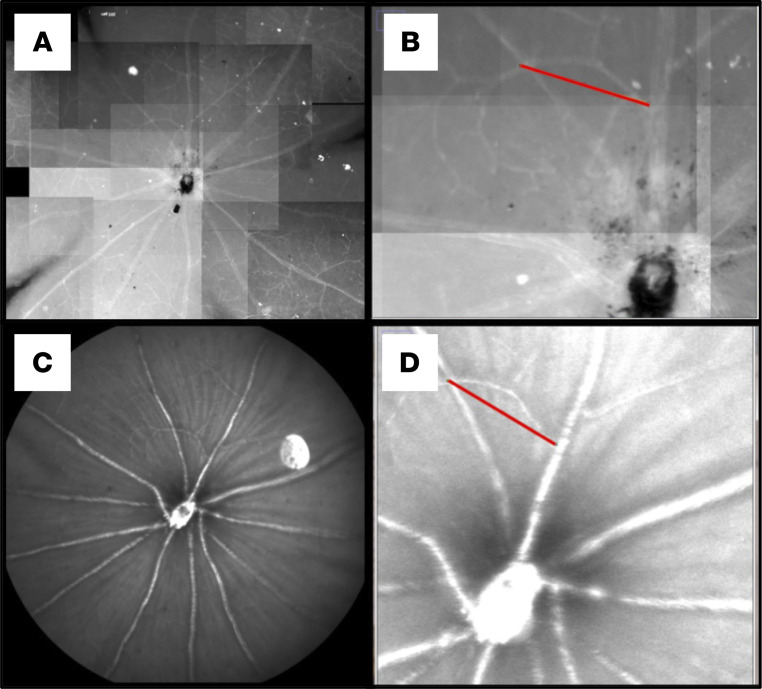
Reliability of CSLO measurements. (**A** and **B**) Microscopy image from a retinal flat mount. (**C** and **D**) The same retina, imaged in vivo through CSLO centered on the optic nerve head, one day prior to eye extraction. The red lines on **B** and **D** show measurements of the same arterial branch in vitro (**B**) and in vivo (**D**).

To assess how CSLO measurements relate with real tissue dimensions, we imaged the retinal vasculature around the optic nerve head of several mice, measuring the distance between given branches or intersections. Following in vivo imaging, mice were sacrificed, and eyeballs extracted as previously described (42). Retinas were then stained with an anti-CD31 (also known as anti–PECAM-1, Abcam, catalog ab28364) antibody. This antibody labels the endothelial cell intercellular junctions, allowing for fluorescence microscopy identification and measurement of the same vascular structures that had been analyzed in vivo. Distances from the same eloquent vascular intersections were measured and compared between in vivo and ex vivo images (Figure 5). Eighty-four distances were measured and compared on CSLO and on the microscope (Nikon E600, Q Capture Pro 7). Comparison of distances between vascular intersections on both in vivo CSLO and matched flat-mount images showed that, in our setting, 1 pixel on a CSLO image corresponds to 1.054 ± 0.099 μm under the fluorescence microscope. Thus, in vivo CSLO measurements are reliable for longitudinal analysis of morphological changes in CX3CR1^GFP/+^ retinae. This is the first study to our knowledge employing CSLO for in vivo immune cell imaging in a MS mouse model validating CSLO as a feasible and reliable technique for the longitudinal analysis of retinal immune cell dynamics in mice expressing fluorescent proteins. Especially in combination with other advanced techniques such as OCT and objective functional measurements (44–46), CSLO empowers the afferent visual system in rodents as a comprehensive model to study the pathophysiology of CNS inflammation (42).

#### Longitudinal retinal CSLO, including acquisition and analysis.

**Figure 6:**
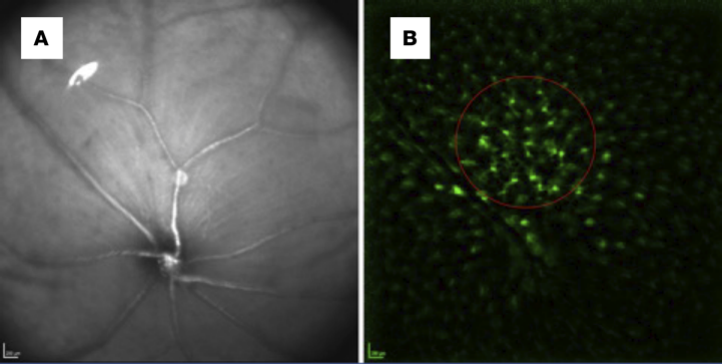
CSLO in CX3CR1^GFP/+^–knock-in mice. (**A**) Infrared fundus image, focused on the retinal nerve fiber layer above the optic disc. (**B**) CSLO imaging of autofluorescent retinal GFP^+^ cells, which in healthy conditions correspond to resting microglia, in CX3CR1^GFP/+^–knock-in mice.

**Figure 7:**
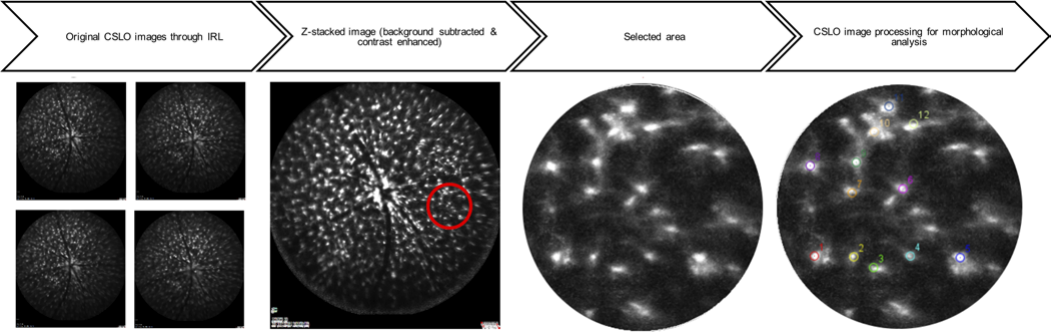
CSLO image processing for the morphological analysis of innate immune cells. The image preparation on ImageJ includes background subtraction (–50 px), contrast enhancement (saturated pixels 1%), and *Z*-stacking after image alignment. Then, a sample area of 350 px (red circle) in the vicinity of the optic nerve head is selected, and the number of cells are measured (somata areas are not shown).

We performed retinal CSLO imaging on 0, 5, 9, 13, 17, 21, 25, 31, 34, 38, 42, 48, and 56 dpi. We used a modified dual-line confocal scanning laser ophthalmoscope with a specialized filter set optimized for this experiment (Spectralis spectral domain–OCT, 488 nm pumped semiconductor laser diode; 500–550 nm bandpass filter to detect GFP). First, the beam was focused on the RNFL layer with the aid of an infrared fundus image (Figure 6). Then, the filter was changed to detect GFP autofluorescence. We obtained images at 4 different focal planes through the IRLs, by adjusting the focus knob. Images were then processed, and the area of interest (defined as a well-focused, 350-pixel area, adjacent to the optic nerve head, while avoiding the major vasculature) was selected for cell count and calculation of the soma area of each cell (Figure 7).

#### Histological analysis and immunofluorescence microscopy.

At day 56 after immunization, mice were sacrificed. We obtained counts of RGC for each retina through in situ immunofluorescence microscopy of retinal whole-mount stained against Brn3a (1:200, Santa Cruz Biotechnology Inc., catalog sc-31984), as described previously (42).

### Transcriptomic changes in retinal microglia during EAE

#### Mice and time points.

Ten-week-old WT female C57BL6/J mice were immunized as described above. Retinal innate immune cells were isolated and pooled for single-cell transcriptomic analysis at 6 (*n =* 21) and 25 (*n =* 19) dpi. In the 25 dpi group, only retinas from mice developing EAE signs (from a total of 25 immunized) were pooled for analysis. Age-matched, nonimmunized female mice (*n =* 20) were used as controls. To avoid any batch effects, all retinas (from control mice and from those immunized 6 and 25 days prior) were extracted, digested, and pooled for analysis on the same day.

#### Isolation of retinal innate immune cells.

Following carbon dioxide euthanasia, the retinae were immediately eviscerated and incubated in papain solution (Worthington Papain Dissociation System) for 90 minutes at 37°C, with gentle pipette mixing every 5 minutes (47). The mixture was then filtered through a 35 μL cell strainer and centrifuged at 300*g* for 5 minutes at room temperature (RT). The cell pellet was then resuspended in a DNase/albumin-inhibitor solution (provided with the dissociation system) and carefully layered over an albumin-inhibitor solution for a discontinuous density gradient by centrifuging at 70*g* for 6 minutes at RT. Cells were then resuspended in a buffer containing phosphate buffered saline (PBS; pH 7.2), 0.5% bovine serum albumin (BSA), and 2 mM EDTA (MACS BSA Stock solution [Miltenyi Biotec, 130-091-376] diluted in autoMACS Rinsing Solution [Miltenyi Biotec, 130-091-222]) at 4°C, incubated initially with an FcR Blocking Reagent, mouse (Miltenyi Biotec, 130-092-575; 15 minutes at 4°C) and subsequently with a PerCP/Cy5-conjugated CD11b anti-mouse/human CD11b antibody (BioLegend, 101228) during 20 minutes at 4°C. After live/dead staining (Invitrogen LIVE/DEAD Fixable Aqua Dead Cell Stain Kit for 405 nm excitation, REF L34957), cells were suspended in the above-mentioned buffer, centrifuged at 70*g* (5 minutes at 4°C), resuspended in buffer and held on ice until sorting.

Cells were sorted using a BD FACS Aria ll with 488 nm laser (for PerCP-Cy5.5, side scatter [SSC], forward scatter [FSC]), FACSDiva v8.0 software, and selecting live single cells based on FSC width, SSC width, and live/dead exclusion. On FSC/SSC plots, live cells were evaluated for PerCP/Cy5-CD11b (Supplemental Figure 2).

### scRNA-seq

#### Next-generation sequencing libraries.

Suspensions of FACS-isolated retinal cells according to size and CD11b staining were then prepared for single-cell isolation and library preparation at the Gladstone Genomics Core facility following the 10x Genomics Cell Preparation Guideline and the instructions for *Preparation of Limited Samples* (48). Next-generation sequencing (scRNA-seq) libraries were obtained for each time-point (6 and 25 dpi, plus nonimmunized controls) using a Chromium Single Cell 3′ Library and Gel Bead Kit (10x Genomics, 120267) and a ChromiumTM Single Cell A Chip Kit (10x Genomics, 1000009).

#### High-throughput single-cell transcriptomic analysis of retinal innate immune cells.

We aligned raw sequence reads and generated gene count matrices using Cell Ranger v2.1.0 (10x Genomics). We performed quality control, clustering, and differential gene expression analysis using Seurat v2.2. (49) We used a total of 10,850 genes across 329 immune cells (total cell count, *n* = 789) for further clustering analysis. Most variable genes were identified using principal component analysis (PCA) and performed t-distributed stochastic neighbor embedding (t-SNE) with significant principal components for clustering and visualization. We identified 17 clusters and defined cell types for each cluster including neuronal, retinal, and immune cells based on marker genes. We confirmed each immune cell type by correlations with public data sets using SingleR software (50). We retrieved immune cell clusters (7 from a total of 17 clusters) and performed clustering analysis within each immune cell population.

### Statistics

All statistical analyses were performed using R version 3.6.1 and Microsoft Excel (51). All results are reported as mean ± SD, if not reported otherwise. *P* < 0.05 were considered significant. To test the reliability of in vivo CSLO versus ex vivo microscopy measurements, we analyzed the variability (mean ± SEM) of the pixel/μm ratios. Cross-sectional and longitudinal within- and between-group analyses, as well as correlations of CSLO, OCT, and histology data, were performed applying mixed linear effect models with accounting for the subject as a random factor. Differentially expressed genes for each immune cell subcluster were identified using Wilcoxon test. Significant genes were selected with 5% of FDR threshold. A functional network and enriched pathways for microglia were generated using *Cytoscape*, including the apps STRING and STRING ENRICHMENT (18, 19). For each time point, transcripts with significantly changed expression (*P <* 0.05) were included in the functional network, and transcripts with a ≥ 2-fold higher or lower expression were selected for enrichment analyses and compared versus the complete mus musculus genome as background using the Reactome Pathways library with a 5% FDR-threshold.

### Study approval

All animal studies were approved by the IRB (AN189496-01A) and performed in accordance to the ARRIVE guidelines.

## Author contribution

ACH, GT, and JHS performed EAE experiments, in vivo imaging, histology, and data postprocessing. JHS designed Figure 5. FCO and KK performed statistical and bioinformatics analyses. FCO and ACH drafted the manuscript. KK performed and analyzed scRNA-seq. EC, BM, and RDS were instrumental in the refining of the ex vivo sample digestion, staining, and FACS protocols. MD, NB, and LR assisted with the image and sample processing. ACH, FCO, KK, CC, SEB, and AJG contributed to data interpretation. ACH, SEB, and AJG designed the research study. SEB and AJG supervised data acquisition, analysis, and manuscript writing. All authors read and approved the final version of the manuscript. Authorship order among co–first authors was assigned alphabetically.

## Supplementary Material

Supplemental data

## Figures and Tables

**Table 1:**
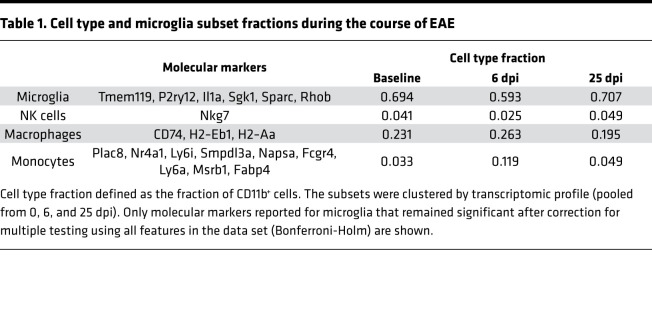
Cell type and microglia subset fractions during the course of EAE

**Table 2:**
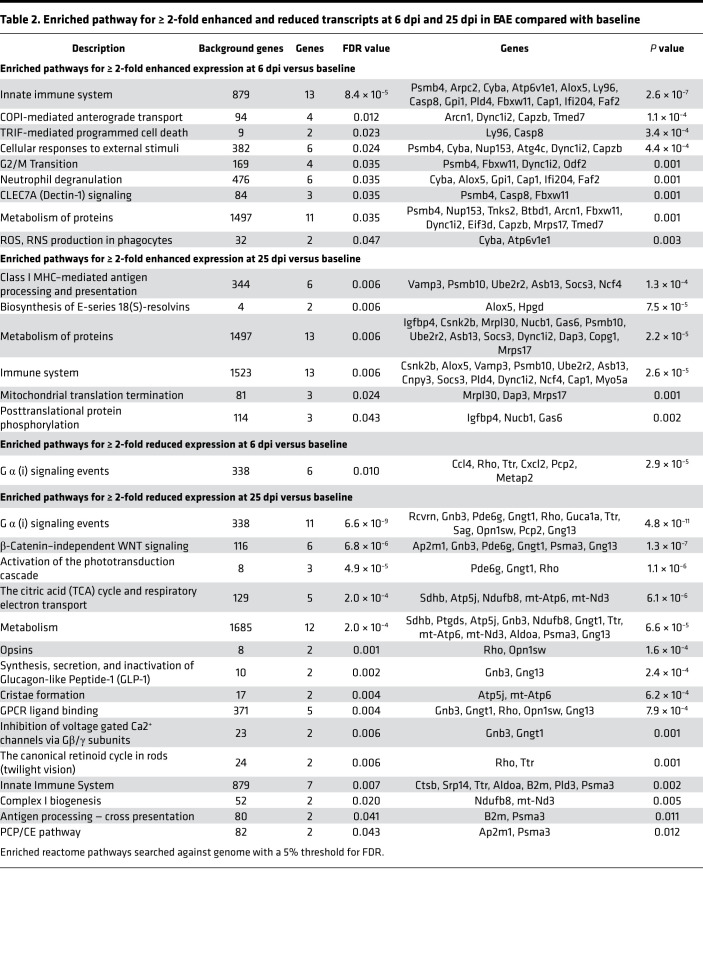
Enriched pathway for ≥ 2-fold enhanced and reduced transcripts at 6 dpi and 25 dpi in EAE compared with baseline

## Version 1 06/08/2021

Electronic publication

## References

[1] Saijo K, Glass CK (2011). Microglial cell origin and phenotypes in health and disease. Nat Rev Immunol.

[2] Lloyd AF (2019). Central nervous system regeneration is driven by microglia necroptosis and repopulation. Nat Neurosci.

[3] Heppner FL (2005). Experimental autoimmune encephalomyelitis repressed by microglial paralysis. Nat Med.

[4] Giunti D (2014). Can we switch microglia’s phenotype to foster neuroprotection? Focus on multiple sclerosis. Immunology.

[5] (2016). Microglial brain region-dependent diversity and selective regional sensitivities to aging. Nat Neurosci.

[6] NBB-Psy (2019). Human microglia regional heterogeneity and phenotypes determined by multiplexed single-cell mass cytometry. Nat Neurosci.

[7] Baker D, Amor S (2014). Experimental autoimmune encephalomyelitis is a good model of multiple sclerosis if used wisely. Mult Scler Relat Disord.

[8] Manogaran P (2019). Retinal pathology in experimental optic neuritis is characterized by retrograde degeneration and gliosis. Acta Neuropathol Commun.

[9] Manogaran P (2018). Exploring experimental autoimmune optic neuritis using multimodal imaging. Neuroimage.

[10] Lloyd AF, Miron VE (2019). The pro-remyelination properties of microglia in the central nervous system. Nat Rev Neurol.

[11] Ajami B (2018). Single-cell mass cytometry reveals distinct populations of brain myeloid cells in mouse neuroinflammation and neurodegeneration models. Nat Neurosci.

[12] Rubino SJ (2018). Acute microglia ablation induces neurodegeneration in the somatosensory system. Nat Commun.

[13] Jin J (2019). Glial pathology and retinal neurotoxicity in the anterior visual pathway in experimental autoimmune encephalomyelitis. Acta Neuropathol Commun.

[14] Seeliger MW (2005). In vivo confocal imaging of the retina in animal models using scanning laser ophthalmoscopy. Vision Res.

[15] Jung S (2000). Analysis of fractalkine receptor CX(3)CR1 function by targeted deletion and green fluorescent protein reporter gene insertion. Mol Cell Biol.

[16] Horstmann L (2016). Microglia response in retina and optic nerve in chronic experimental autoimmune encephalomyelitis. J Neuroimmunol.

[17] Nissen JC (2018). Csf1R inhibition attenuates experimental autoimmune encephalomyelitis and promotes recovery. Exp Neurol.

[18] Shannon P (2003). Cytoscape: a software environment for integrated models of biomolecular interaction networks. Genome Res.

[19] Doncheva NT (2019). Cytoscape StringApp: network analysis and visualization of proteomics data. J Proteome Res.

[20] Brown GD (2006). Dectin-1: a signalling non-TLR pattern-recognition receptor. Nat Rev Immunol.

[21] Simpson DSA, Oliver PL (2020). ROS generation in microglia: understanding oxidative stress and inflammation in neurodegenerative disease. Antioxidants (Basel).

[22] Rey C (2016). Resolvin D1 and E1 promote resolution of inflammation in microglial cells in vitro. Brain Behav Immun.

[23] Halleskog C (2011). WNT signaling in activated microglia is pro-inflammatory. Glia.

[24] Reactome. PCP/CE Pathway. https://reactome.org/content/detail/R-HSA-4086400 Updated August 8, 2013. Accessed February 4, 2021

[25] Gruber RC (2014). Targeted GAS6 delivery to the CNS protects axons from damage during experimental autoimmune encephalomyelitis. J Neurosci.

[26] Tsiperson V (2010). GAS6 enhances repair following cuprizone-induced demyelination. PLoS One.

[27] Binder MD (2011). Gas6 increases myelination by oligodendrocytes and its deficiency delays recovery following cuprizone-induced demyelination. PLoS One.

[28] Sainaghi PP (2013). Growth arrest specific gene 6 protein concentration in cerebrospinal fluid correlates with relapse severity in multiple sclerosis. Mediators Inflamm.

[29] Li Y (2006). Dendritic cells transduced with SOCS-3 exhibit a tolerogenic/DC2 phenotype that directs type 2 Th cell differentiation in vitro and in vivo. J Immunol.

[30] Liu X (2008). Loss of STAT3 in CD4+ T cells prevents development of experimental autoimmune diseases. J Immunol.

[31] Emery B (2006). SOCS3 negatively regulates LIF signaling in neural precursor cells. Mol Cell Neurosci.

[32] Kong W (2016). The natural dual cyclooxygenase and 5-lipoxygenase inhibitor flavocoxid is protective in EAE through effects on Th1/Th17 differentiation and macrophage/microglia activation. Brain Behav Immun.

[33] Yoshikawa K (2011). Inhibition of 5-lipoxygenase activity in mice during cuprizone-induced demyelination attenuates neuroinflammation, motor dysfunction and axonal damage. Prostaglandins Leukot Essent Fatty Acids.

[34] Emerson MR, LeVine SM (2004). Experimental allergic encephalomyelitis is exacerbated in mice deficient for 12/15-lipoxygenase or 5-lipoxygenase. Brain Res.

[35] Miron VE (2013). M2 microglia and macrophages drive oligodendrocyte differentiation during CNS remyelination. Nat Neurosci.

[36] Miron VE (2017). Microglia-driven regulation of oligodendrocyte lineage cells, myelination, and remyelination. J Leukoc Biol.

[37] Magon S (2020). Volume loss in the deep gray matter and thalamic subnuclei: a longitudinal study on disability progression in multiple sclerosis. J Neurol.

[38] Zhang J (2021). Gray matter atrophy cannot be fully explained by white matter damage in patients with MS. Mult Scler.

[39] Lombardi M (2019). Detrimental and protective action of microglial extracellular vesicles on myelin lesions: astrocyte involvement in remyelination failure. Acta Neuropathol.

[40] (2018). Rapid and integrative discovery of retina regulatory molecules. Cell Rep.

[41] Spangenberg E (2019). Sustained microglial depletion with CSF1R inhibitor impairs parenchymal plaque development in an Alzheimer’s disease model. Nat Commun.

[42] Cruz-Herranz A (2019). Monitoring retinal changes with optical coherence tomography predicts neuronal loss in experimental autoimmune encephalomyelitis. J Neuroinflammation.

[43] Cruz-Herranz A (2016). The APOSTEL recommendations for reporting quantitative optical coherence tomography studies. Neurology.

[44] Hecker C (2020). Comparison of different optomotor response readouts for visual testing in experimental autoimmune encephalomyelitis-optic neuritis. J Neuroinflammation.

[45] Dietrich M (2019). Using Optical Coherence Tomography and Optokinetic Response As Structural and Functional Visual System Readouts in Mice and Rats. J Vis Exp.

[46] Schirmer L (2018). Oligodendrocyte-encoded Kir4.1 function is required for axonal integrity. Elife.

[47] Skeie JM (2011). Evisceration of mouse vitreous and retina for proteomic analyses. J Vis Exp.

[48] 10x Genomics. *Single Cell Protocols — Cell Preparation Guide.* 10x Genomics, Inc; 2017.

[49] Butler A (2018). Integrating single-cell transcriptomic data across different conditions, technologies, and species. Nat Biotechnol.

[50] Aran D (2019). Reference-based analysis of lung single-cell sequencing reveals a transitional profibrotic macrophage. Nat Immunol.

[51] R Core Team. *R: A Language And Environment For Statistical Computing.* R Foundation for Statistical Computing; 2018:

