# Supplementary material for: Distinctive waves of innate immune response in the retina in experimental autoimmune encephalomyelitis
Source: JCI Insight. 2021 Jun 8;6(11):e149228. doi: 10.1172/jci.insight.149228 (PMC8262300; doi:10.1172/jci.insight.149228)
Supplement: Supplemental data [file jciinsight-6-149228-s213.pdf]

Supplement

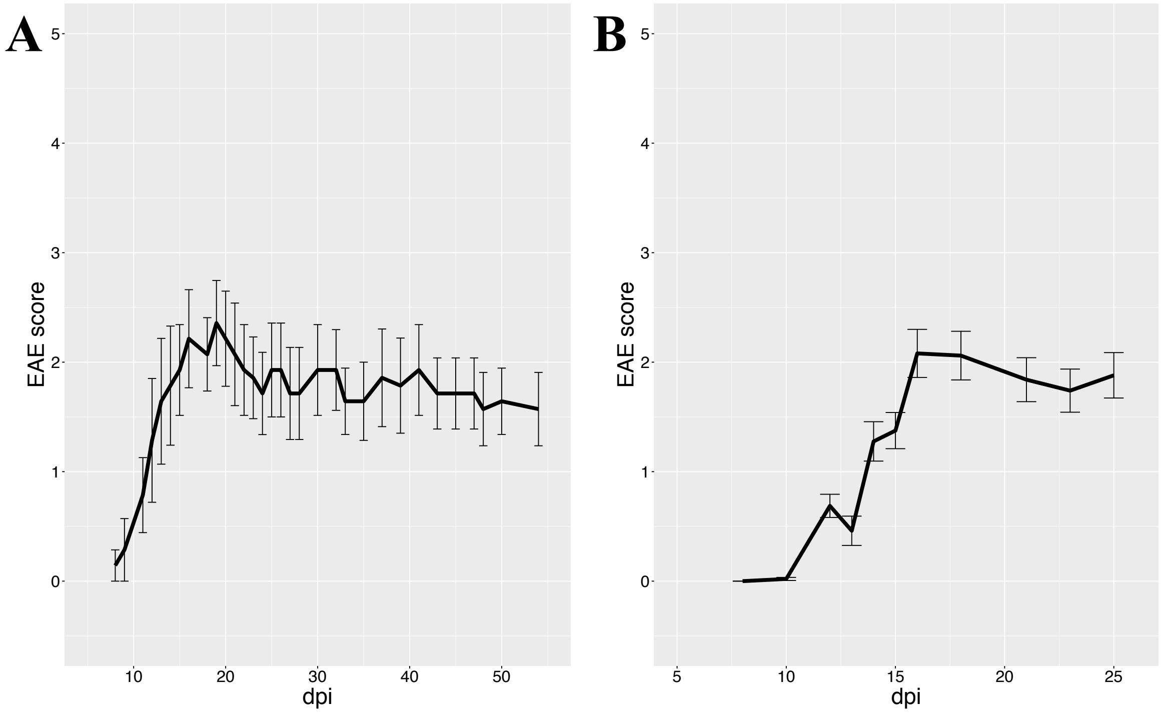

Supplementary figure 1: EAE scores for mice included in A) longitudinal imaging (CSLO, OCT) and histopathological analyses and B) genomics analyses. All results displayed as mean ± standard error.

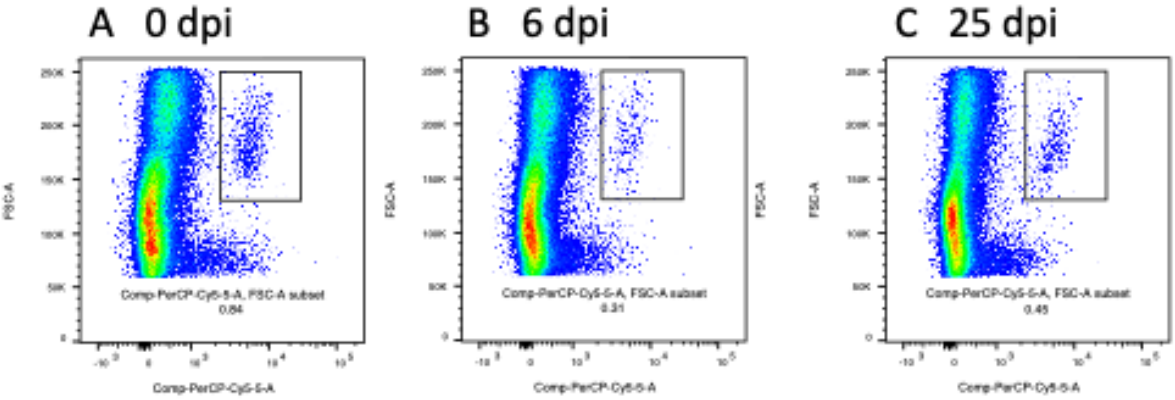

Supplementary figure 2: Cells were sorted by FACS and live single cells were selected based on forward scatter (FSC) width, side scatter (SSC) width, and live/dead exclusion. On FSC/SSC plots, live cells were evaluated for PerCP/Cy5-CD11b.
